# Analysis of advice and guidance primary care queries to rheumatology over a calendar year—a diagnostic service evaluation

**DOI:** 10.1093/rap/rkag054

**Published:** 2026-05-20

**Authors:** Ethan O’Shaughnessy, Ben Mulhearn, Marwan Bukhari

**Affiliations:** Lancaster Medical School, Lancaster University, Lancaster, UK; Department of Rheumatology, University Hospitals of Morecambe Bay NHS Foundation Trust, Royal Lancaster Infirmary, Lancaster, UK; Lancaster Medical School, Lancaster University, Lancaster, UK; Department of Rheumatology, University Hospitals of Morecambe Bay NHS Foundation Trust, Royal Lancaster Infirmary, Lancaster, UK

**Keywords:** qualitative, rheumatology, advice and guidance, health service improvement, service evaluation

## Abstract

**Objectives:**

In the UK, advice and guidance services allow general practitioners (GPs) to seek consultant assistance without formal referral, supporting decision-making and reducing unnecessary secondary care referrals. However, a significant proportion of advice and guidance queries represent frequently asked questions (FAQs), placing avoidable burden on consultants and delaying patient care while replies are awaited. This diagnostic service evaluation aimed to identify common FAQs within rheumatology advice and guidance queries at Morecambe Bay Hospitals Trust (MBHT), with the goal of developing targeted educational material for GPs. This material seeks to improve the quality of advice and guidance referrals to reduce unnecessary secondary care attendances and enhance the delivery of urgent referral pathways in line with targets that are proven to improve patient outcomes. The methodology presented in this article is a reproducible method to bring about iterative improvements to referrals to an individual department through education and behaviour change.

**Methods:**

We conducted a retrospective, qualitative content analysis of 2499 GP‑initiated rheumatology advice and guidance messages submitted between April 2024 and April 2025. Our analysis occurred in three stages. First, we used NVivo 15 to perform a word‑frequency analysis to identify common, clinically significant phrases within advice and guidance queries. Next, for each often‑occurring term in our frequency map, we drew a purposive sample of the full advice and guidance conversation in which it appeared. From these, we conducted an in‑depth, iterative coding of their content—identifying and refining subthemes—until no new themes were identified. Finally, we filtered the dataset for messages containing ‘urgent’, ‘acute’, or ‘emergency’ to isolate time‑sensitive queries.

**Results:**

Three major themes of FAQs were identified: interpretation of blood tests (especially incongruous blood results and the significance of inflammatory markers), management of osteoporosis (particularly medication queries) and initial treatment or diagnostic uncertainty in suspected giant cell arteritis (GCA). Queries marked as urgent were most commonly associated with GCA. The GPs’ questions were frequently similar within their themes and amenable to concise, standardised advice.

**Conclusion:**

Recurring patterns in advice and guidance queries suggest an opportunity to support local GPs through focused educational material; a guidance document targeting the most common FAQs is in development. Although implementation and evaluation are pending, this low-cost, scalable approach may feasibly offer value across specialties and sites. Further study will assess its impact on advice and guidance usage and GP confidence.

Key messages

- Our diagnostic service evaluation identified frequently asked questions within our received advice and guidance queries that are amenable to education to improve the quality of referrals.
- Our qualitative approach is appropriately thorough to identify such findings but flexible and straightforward enough to be adapted and reproduced by different healthcare departments to bring about health service improvements.

## Introduction

In the UK, advice and guidance services provide a platform for general practitioners (GPs) to seek timely advice from specialist teams. These services are a key component of the National Health Service Long Term Plan, aiming to streamline care provision and reduce unnecessary referrals [1]. At Morecambe Bay Hospitals Trust (MBHT), advice and guidance has been in place since 2001, enabling GPs and consultants to communicate via a browser-based, text message–style interface.

Despite the value of advice and guidance in supporting clinical decision-making, a significant proportion of queries involve frequently asked questions (FAQs). Responding to these recurring queries places a repeated administrative burden upon consultants, delaying patient care and creating frustration. This diagnostic service evaluation pilot project seeks to address this issue by identifying GPs’ FAQs and co-developing an advice document to pre-emptively answer them. The methodology in this article could be reproduced within individual departments to improve their unique referral pathways through improved education and behaviour change in referrers.

While recent quality improvement efforts in rheumatology have increasingly focused on process optimisation and the use of information technology (IT) [2], to our knowledge there are no published diagnostic service evaluation initiatives surrounding advice and guidance within rheumatology. In other specialties, and more generally, there exists much research regarding the utility of advice and guidance, but no service evaluations exist targeting FAQs to bring about iterative improvement in referrals.

## Methods

### Design

We conducted a retrospective, qualitative content analysis of advice and guidance messages as part of a diagnostic service evaluation project aimed at reducing FAQs. This article follows SQuIRE 2.0 Guidelines [3], adapted to its format as a pilot project.

### Data collection

An exhaustive list of advice and guidance messages between GPs and the MBHT rheumatology department was retrospectively extracted from the advice and guidance system. It included all messages exchanged between April 2024 and April 2025.

### Sample

In the MBHT rheumatology department, all advice and guidance queries are submitted by GPs and answered by consultants. Inclusion criteria were messages within the conversation were clinical in nature and the conversation was initiated by a GP. Incomplete, administrative and duplicate entries were excluded. From an initial 2502 messages, 2499 (99.9%) were included within the analysis. Greater analytical focus was placed on the initial GP query messages (as opposed to subsequent correspondence), of which there were 1016 (40%).

### Ethical considerations

This project was not considered research and thus did not require committee approval [5]. The viewing of any potentially confidential information contained within advice and guidance queries was only undertaken by the authors of this article who were directly involved in patient care and all data were stored securely using hospital computers. The data were initially collected by the hospital IT and data department. Anonymisation was not required as the dataset provided did not include patient details attached to the referral and GPs did not include them in the body of their questions.

### Data analysis

We used a pragmatic, mixed methods approach combining word frequency analysis with targeted thematic review:

#### Stage 1: word frequency mapping

To support theme generation, we used NVivo 15 (Lumivero, Denver, CO, USA) [4] software to perform a basic word frequency analysis of the advice and guidance dataset. Common stop words (e.g. ‘the’, ‘and’, ‘then’) were excluded from the output. The resulting list was then manually reviewed to extract clinically relevant terms and phrases. These were grouped into conceptually related categories—such as ‘medications’ and ‘blood tests’—to inform initial coding. Variations of the same root word (e.g. ‘hand’ and ‘hands’) were counted once, while typographical errors and nonsensical entries (e.g. ‘hnads’) were excluded. This latter exclusion was to avoid error in their interpretation, as word entries appeared without context in the frequency map. The list was reviewed until words represented <0.03% of word appearances, at which point the majority of items were nonsensical misspellings.

#### Stage 2: targeted review

Building on our initial codes, we identified the highest‐frequency terms within each category and, for each, extracted a purposive sample of the complete advice and guidance messages containing that word. These messages were then analysed in depth, iteratively refining subthemes, until successive rounds yielded no new themes (thematic saturation). This process ensured that our final themes fully captured the scope of GP queries.

The major FAQs were identified during this stage of the analysis and were reviewed with the consultant team to ensure they were both perceived as clinically significant and amenable to the creation of an advice sheet.

#### Stage 3: keyword filtering

All messages containing the word ‘urgent’, ‘emergency’ and ‘acute’ were separately analysed to capture themes that GPs perceived as especially time-sensitive.

### Intervention

The FAQs identified by the analysis were presented to the rheumatology multidisciplinary team and an advice sheet targeting them was created by the consultant body. It is due to be finalised and distributed to local GPs at the time of writing.

### Reanalysis

A follow-up analysis is planned 1 year after the advice sheet’s implementation, including analysis of the total number of queries and their content, to evaluate the material’s impact. Feedback will also be collected from both the consultant team and local GPs to inform further development of the resource.

### Reflexivity and rigour

Analysis was led by a fifth-year medical student with qualitative research experience and reviewed by a supervising consultant. Although formal double coding was not used, regular peer discussion was used to improve coding consistency and reduce individual bias. This approach was appropriate to the scope and aims of a local diagnostic service evaluation project.

## Results

### Review of the dataset

Analysis included 1016 GP‑initiated conversations comprising 2499 messages (April 2024–April 2025).

### Initial themes

Word frequency analysis identified six broad themes that recurred across GP queries (Table 1).

**Table 1:** Initial themes after word frequency analysis.

Initial theme	Examples	Frequency of word use within theme, *n*
Anatomical	Spine, hand	904
Blood results	CRP, ESR	1973
Clinical features	Swollen, headache	1004
Clinical entities	Osteoarthritis, SLE	655
Imaging	DEXA, erosion	457
Medications	Denosumab, NSAID	1231

The ‘anatomical’ category captured mentions of body sites—most frequently ‘joint’ (*n* = 192), ‘hand’ (*n* = 138) and ‘knee’ (*n* = 127). GPs often noted these areas for context, and there were no recurring questions by body site that could inform targeted educational material.

The ‘blood results’ category was the most frequently utilised theme, with 1973 word instances. The most common words within the group were ‘bloods’ (*n* = 397), ‘ESR’ (*n* = 275) and ‘t’ [*sic*] (*n* = 275). NVivo’s word‑frequency tool treats each space‑delimited entry separately, so the phrase ‘t value’ appears as two items: ‘t’ and ‘value’. To clarify its meaning, we randomly sampled 50 instances of ‘t’ (out of 275) and found 49 were associated with the phrase ‘t value’ (the lone exception being a typing error). We undertook a similar investigation for various unclear phases throughout the analysis. Beyond this, GPs also frequently queried C-reactive protein (CRP) (*n* = 201), vitamin D levels (*n* = 189), white cell count (WCC) (*n* = 65) and anti-nuclear antibodies (ANA) (*n* = 47).

The ‘clinical features’ category was dominated by ‘swelling’ (*n* = 182), ‘stiffness’ (*n* = 145) and ‘pain’ (*n* = 116), which together accounted for 44% of word uses in this group. A further 13% comprised ‘visual’ (*n* = 32), ‘temporal’ (*n* = 43) and ‘headache’ (*n* = 55), underscoring GP concerns about GCA. Although ‘temporal’ is semantically anatomical, it appears within clinical features due to its function as an adjective of GCA symptoms. While none of these terms is exclusive to GCA—‘headache’ being especially non-specific—a targeted sample confirmed they were overwhelmingly utilised within the context of suspected GCA.

The ‘clinical entities’ category highlighted ‘osteoporosis’ (*n* = 171), ‘rheumatoid’ [arthritis] (*n* = 146) and ‘osteoarthritis’ (*n* = 85) as the most commonly queried conditions. Again, ‘GCA’ was a significant entry representing 8% of the word uses within this category. Other significant conditions within the data included ‘lupus’ (*n* = 33), ‘psoriasis’ (*n* = 30) and ‘gout’ (*n* = 62).

The ‘imaging’ theme encompassed both modalities and findings (e.g. ‘erosion’) but was driven almost entirely by DEXA (*n* = 260), which accounted for 57% of word use. Our focused review showed that GPs were not asking about scan technique or interpretation, rather they tended to cite DEXA results as context when querying osteoporosis treatments.

The ‘medications’ category comprised 1231 word instances, 26% of which were ‘steroid’ (*n* = 169) or ‘prednisolone’ (*n* = 148). Further sampling revealed these terms were usually cited simply to describe a patient’s existing therapy. Most medication questions instead concerned osteoporosis treatments. Indeed, ‘acid’ (*n* = 178) (from ‘alendronic acid’ and ‘zoledronic acid’) was the single most frequent word in this group.

Finally, among entries containing ‘urgent’ (*n* = 63), ‘emergency’ (*n* = 2) or ‘acute’ (*n* = 59), the vast majority related to suspected GCA cases. Other notable subthemes included GPs seeking guidance on managing disease flares and asking what interim steps to take after making an urgent referral.

### Final themes

Following an analysis of purposively selected samples of complete queries derived from the initial categories outlined above, three major themes of FAQs were synthesised and identified as focuses for the advice sheet (Table 2).

**Table 2:** The major types of FAQs.

Theme of FAQ	Illustrative example
The significance of blood results	I have a patient with a positive rheumatoid factor but symptoms otherwise osteoarthritic in nature, should I refer to rheumatology?
Osteoporosis medication queries	A patient has numerous contraindications to bisphosphonates and denosumab, is there a further agent you could recommend?
GCA	I am worried about my 76-year-old patient with a unilateral headache. She has PMR and an ESR of 54 but her headache responds to typical analgesia. Is it likely to be GCA?

The most common FAQ theme was related to the interpretation of blood tests. GPs queried elevated blood tests and immunological markers in patients who were otherwise well. Additionally, GPs asked about rheumatological symptoms alongside negative immunology. Indeed, ‘positive’ (*n* = 81) and ‘negative’ (*n* = 89) appeared in a similar number of entries. Another major FAQ theme related to inflammatory markers; GPs often enquired about how high CRP, ESR or white cell count had to be in order to be of concern. Patients with borderline results were asked about most commonly; patients with clearly abnormal results were referred directly without query.

The next most frequent query type regarded osteoporosis treatment. GPs were unsure how to proceed with conflicting data (e.g. normal DEXA but a history of fragility fracture) and lacked confidence prescribing second‑line agents when first‑line therapies were contraindicated or not tolerated. Questions within this theme were context‑rich, often accompanied by detailed patient histories.

Although fewer in number, queries related to GCA were often the most urgent, with GPs stressing the concerning nature of patients’ clinical condition. Questions related to both the likelihood of GCA given a patient’s history and to the initial management when a referral had already been made. An especially common query was whether it was appropriate to prescribe a higher dose of steroid. Retrospective queries were most commonly seen in relation to GCA, where GPs had often already initiated treatment prior to messaging.

## Discussion

### Key findings

Analysis of advice and guidance queries revealed three major recurring themes in FAQs that aligned closely with those predicted by the consultant body prior to review. The presence of consistently similar questions highlights a potential gap within the experience of local GPs to confidently and independently manage suspected GCA, complex osteoporosis medications and seemingly incongruous blood results. Questions frequently required simple, direct answers and were frequently similar across related queries, making them suitable for a concise guidance document.

There exists national and local guidance related to our three major themes, yet the FAQs suggest that these are either unknown or inaccessible to GPs. If the advice sheet was to be implemented directly within the local advice and guidance referral pathway this would feasibly increase engagement, and continual feedback and review will allow the sheet to be targeted towards the evolving needs of GPs in the area.

It is important to mention that repeated questions were asked by different GPs at different practices and repeated throughout the calendar year. This suggests that questions are likely to represent gaps in GP knowledge as a whole and not a deficiency within an individual practice or professional.

### Strengths

The principal strength of this article lies in the flexibility and transferability of the methodology as part of service evaluation. Word frequency analysis provided a broad overview of commonly used terms, ensuring that high-volume topics were not overlooked. However, word frequency analysis alone does not capture clinical nuance or user intent. To address this limitation, purposive sampling of high-frequency terms enabled in-depth qualitative analysis, allowing exploration of the context and meaning underlying frequently used language. This two-stage approach ensured that the advice sheet was informed not only by volume but also by clinical relevance, thereby enhancing its practical utility. A third stage, utilising keyword filtering, enabled the identification of particularly urgent queries within the sample. Collectively, these stages are general enough to feasibly be adapted and applied across different healthcare departments while maintaining their intended methodological thoroughness.

Another strength of this project was the large dataset, encompassing all rheumatology advice and guidance queries submitted over a full calendar year. This allowed for a comprehensive and longitudinal view of real-world GP behaviours, concerns and decision-making. The volume and time span of the data enhanced the generalisability and robustness of the findings, which would allow it to capture potential seasonal variation and recurring themes not evident in smaller or cross-sectional samples.

### Limitations

The major limitation of this article is that it is based within an individual department with its own unique referral system that is unlikely to be the same across other healthcare departments. For example, in MBHT rheumatology referrals are received only from GPs and answered only by consultants. It is feasible that departments that receive and answer queries written by other healthcare professionals would have unique findings. Yet, the flexibility of the methodology would allow it to be responsive to these difference if executed in such departments.

GPs may feel that advice and guidance messaging provides personalised and less generalisable advice than traditional written guidance. Similarly, it may be felt that advice and guidance is an easier and more direct method of seeking assistance, which provides a more definitive answer with shared responsibility. However, the aim of the project is not to replace advice and guidance, but to improve its use and provide education.

A further limitation of this project is that it did not include formal double coding or intercoder reliability measures, which could be used to enhance objectivity. Instead, coding and theme development were carried out collaboratively within a small project team, with regular discussion to ensure consistency and clarity. While this approach may introduce some subjectivity, it was appropriate for the scale and aims of a diagnostic service evaluation rather than an intensely rigorous research project. The goal was not theoretical generalisability, but to generate practical, actionable insights to inform an intervention in a specific context. This does, however, partially limit the project’s application to other centres.

Finally, our approach to word‑frequency mapping, while effective for highlighting broad patterns, does not yield precise quantitative counts. By consolidating related tokens (e.g. ‘hand’ and ‘hands’) into a single entry and excluding nonsensical terms, we inevitably underrepresent the true frequency of some concepts. Consequently, the actual number of queries pertaining to any given theme is likely higher than our estimates suggest. Moreover, because we purposively sampled threads for in‑depth review rather than exhaustively coding every message, we cannot state exact totals for each major FAQ category. In practice, even if the guidance document succeeds in reducing FAQ‑related submissions, the overall impact on consultant workload may be modest if the absolute number of queries within each theme is relatively small.

### Next steps

Following implementation, we plan to carry out an analysis to assess the impact of the material, including assessment of:

- Query volume: we will measure total advice and guidance rheumatology queries monthly, aiming for a reduction in GCA, osteoporosis and blood result FAQs over 12 months. In parallel, consultant opinion on the nature of the advice and guidance workload is to be assessed.
- Engagement with material: we will review a random sample of queries to assess whether more cases could have been answered using the advice sheet.
- GP confidence and satisfaction: we will deploy a survey at 6 and 12 months post‑implementation, capturing GP self‑reported confidence (5‑point Likert scale) in managing GCA, osteoporosis and borderline blood results before and after using the guidance.
- Subjective patient experience: we will review the subjective satisfaction of patients’ experience with the existing referral pathway using a 10-point scale and compare these to a further sample after implementation of the advice sheet.
- Enhancement of timely referrals: we will monitor how many patient referrals are being achieved in a timely manner and assess whether improvements are achieved in this domain.

Future iterations of the tool may involve co-production with GPs and integration into the electronic referral interface to enhance accessibility and uptake.

## Conclusions

This diagnostic service evaluation identified clear patterns in rheumatology advice and guidance queries, with a small number of recurring themes—blood test interpretation, osteoporosis treatment and suspected GCA—accounting for a proportion of repeated messages. These findings highlight an opportunity to improve efficiency and support GP decision-making through targeted educational materials. While the advice sheet has yet to be implemented, its development is grounded in a robust analysis of real-world queries and close consultant involvement, suggesting strong potential for utility. If effective, it could reduce repetitive messaging, streamline care and enhance GP confidence in key areas of uncertainty.

The approach taken—a qualitative review of advice and guidance data—offers a low-cost, scalable model that could be replicated in other specialties and settings. Its sustainability depends on ongoing review and integration into digital workflows, which are planned as part of future development. Next steps include implementing the advice sheet, evaluating its impact on query volume and GP engagement and gathering user feedback. Further study will help determine how such tools can be co-produced, embedded into routine practice and adapted across specialties to support safe, efficient care delivery.

## Data Availability

The data supporting the findings of this study are confidential and cannot be made publicly available due to privacy and ethical restrictions.
